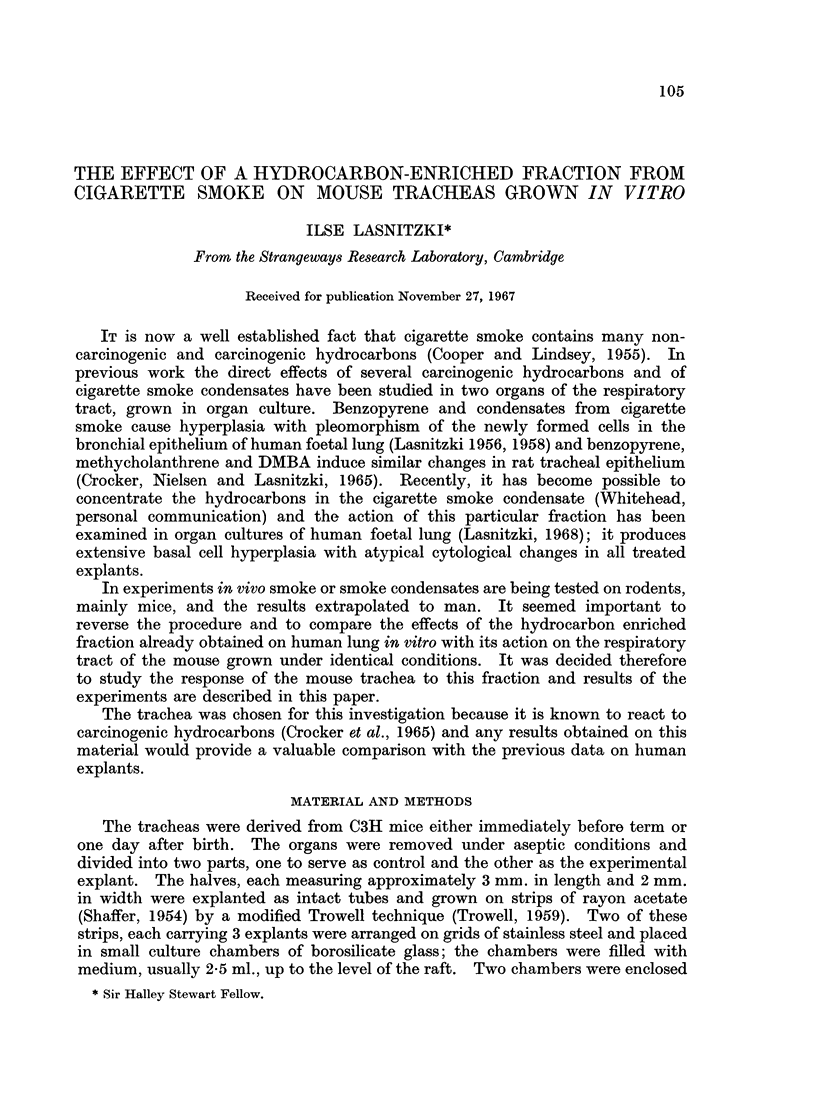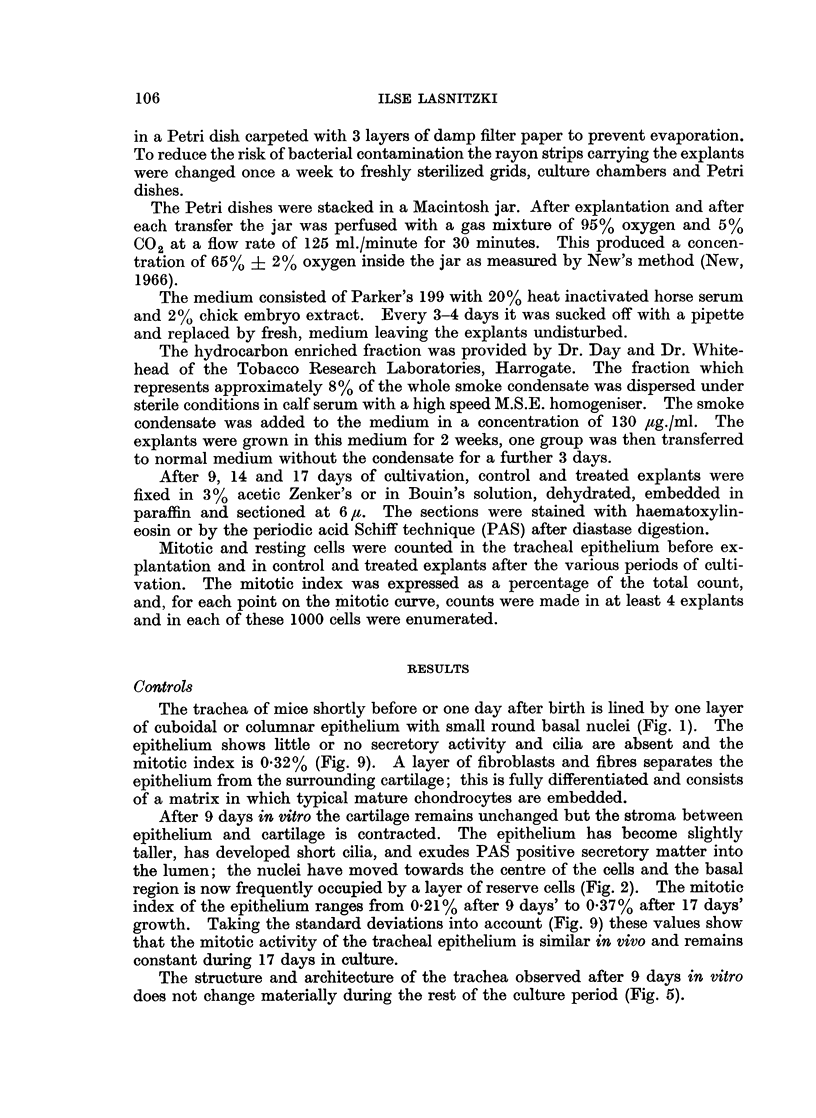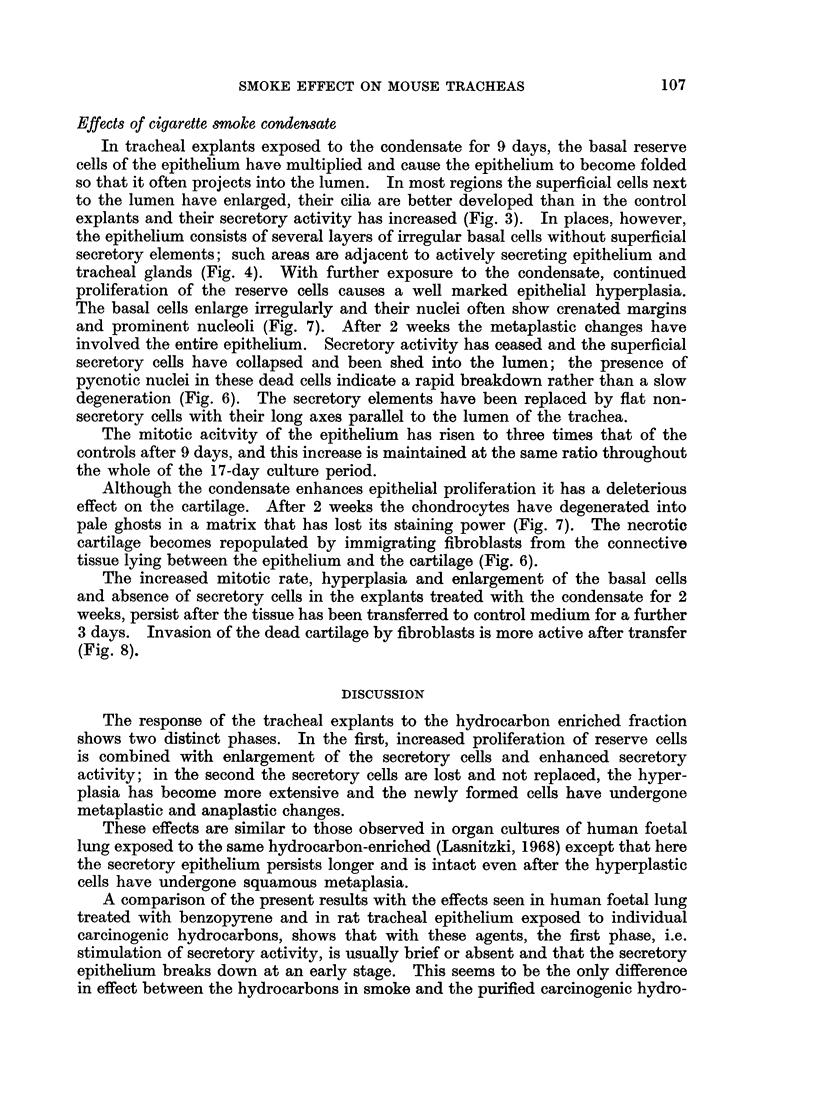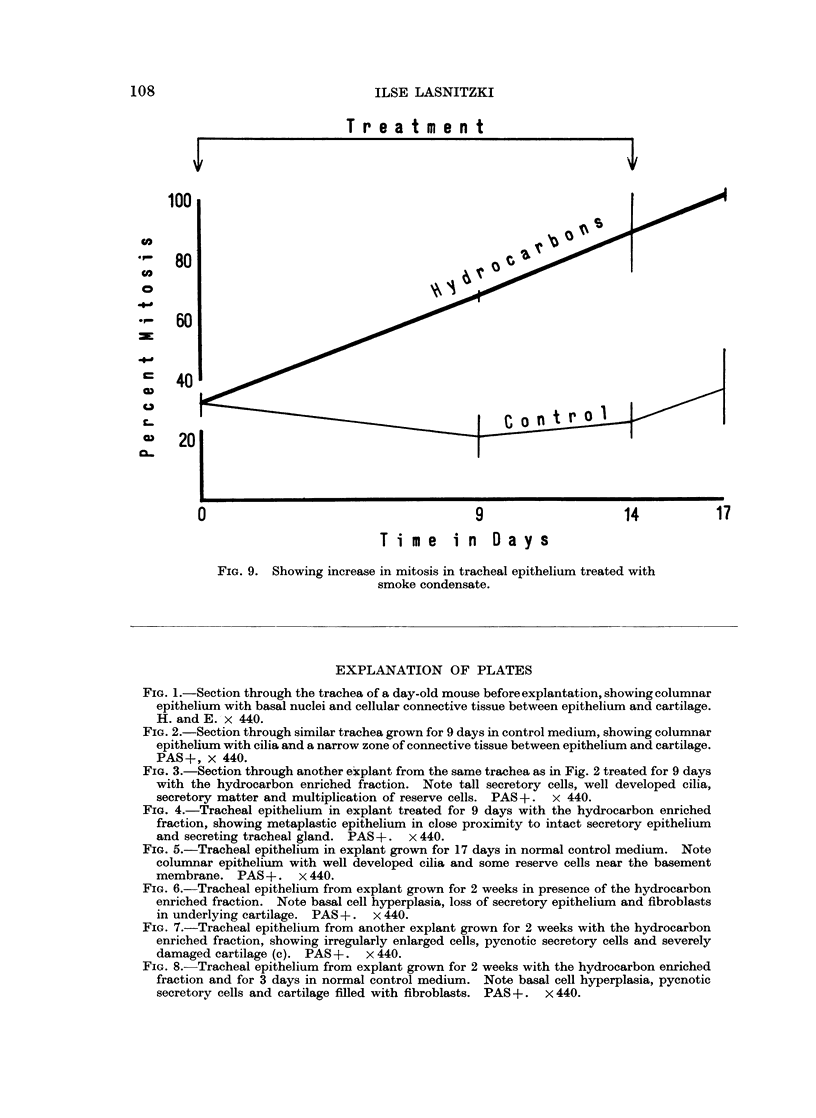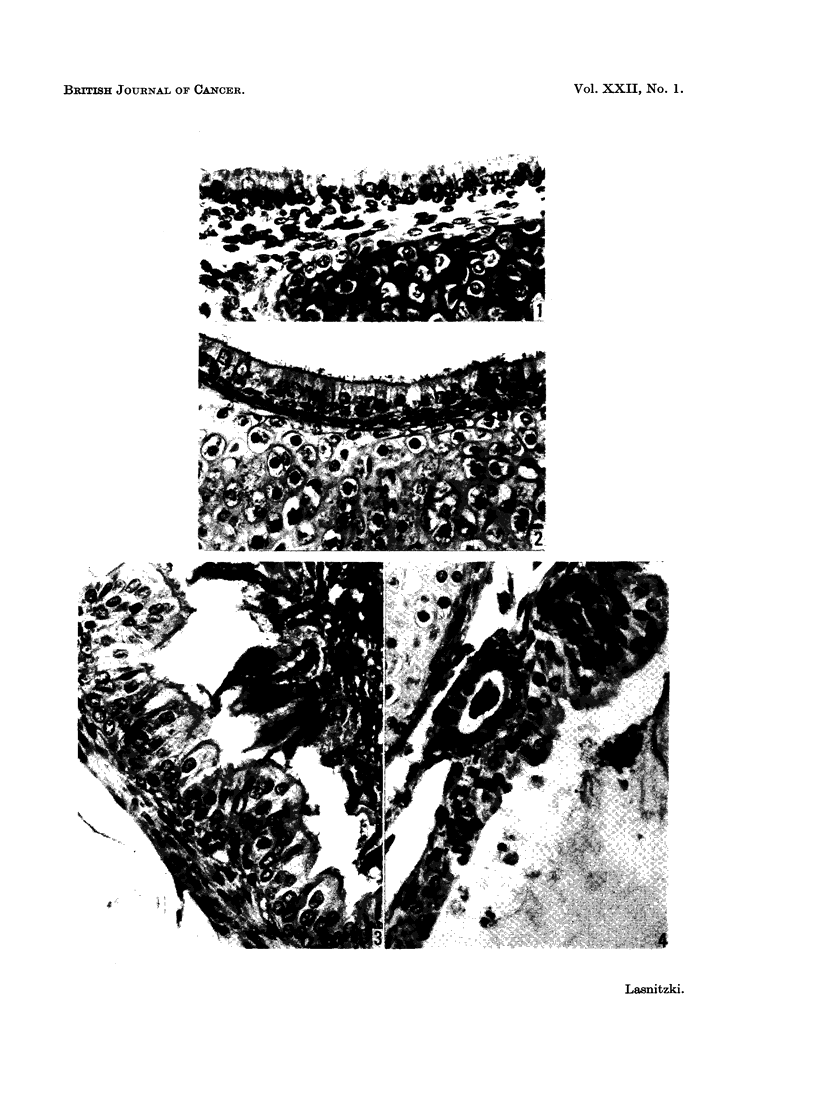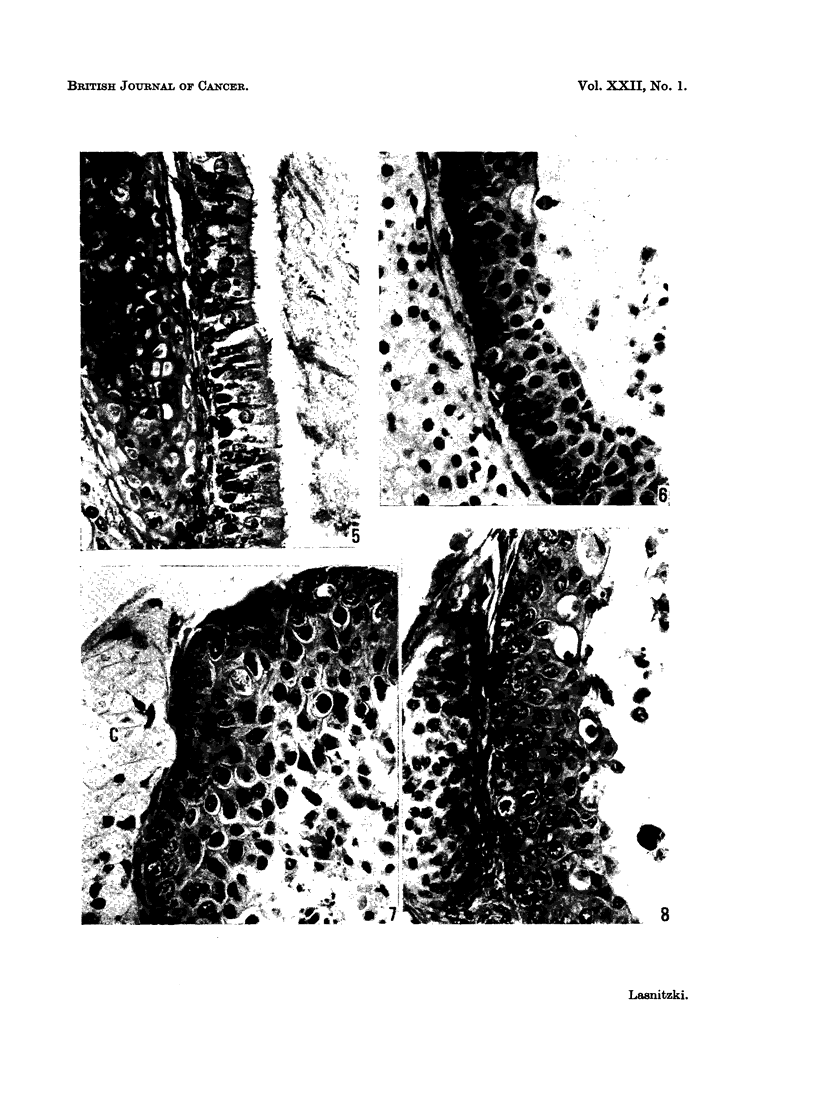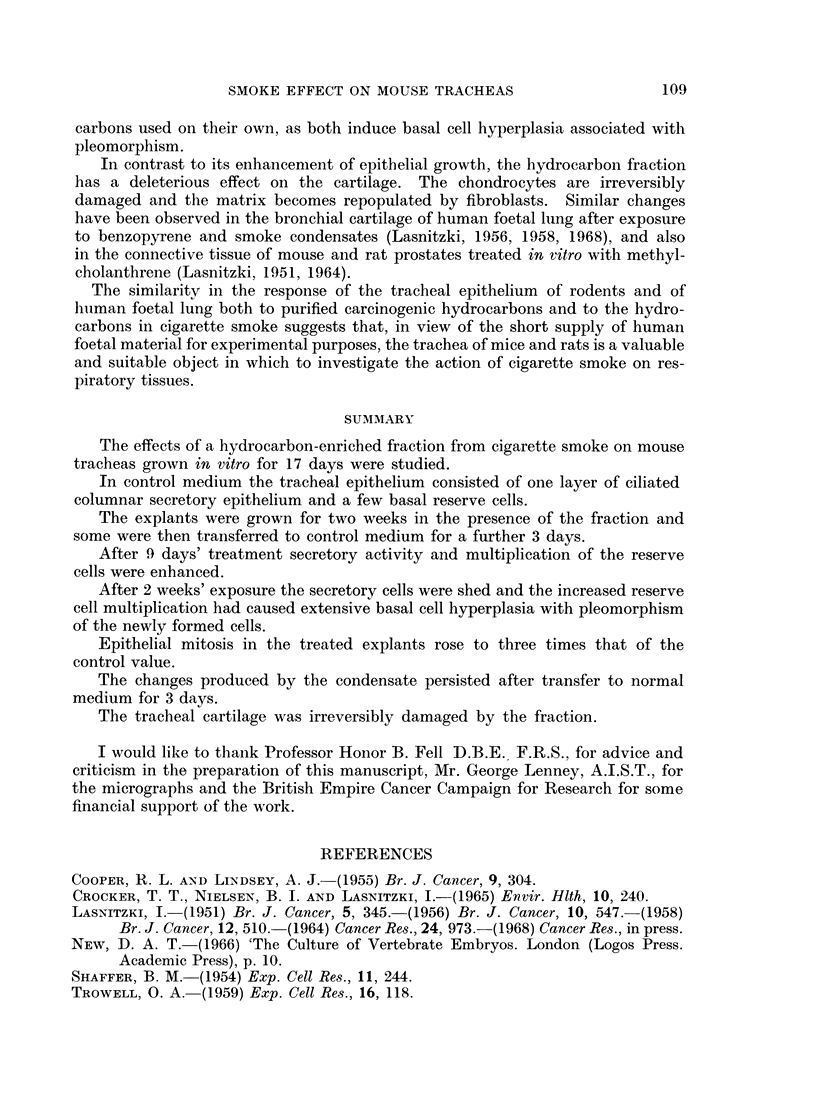# The effect of a hydrocarbon-enriched fraction from cigarette smoke on mouse tracheas grown in vitro.

**DOI:** 10.1038/bjc.1968.14

**Published:** 1968-03

**Authors:** I. Lasnitzki

## Abstract

**Images:**


### THE EFFECT OF A HYDROCARBON-ENRICHED FRACTION FROM CIGARETTE SMOKE ON MOUSE TRACHEAS GROWN *IN VITRO*

ILSE LASNITZKI*

*From the Strangeways Research Laboratory, Cambridge*

Received for publication November 27, 1967

IT is now a well established fact that cigarette smoke contains many non-carcinogenic and carcinogenic hydrocarbons (Cooper and Lindsey, 1955). In previous work the direct effects of several carcinogenic hydrocarbons and of cigarette smoke condensates have been studied in two organs of the respiratory tract, grown in organ culture. Benzopyrene and condensates from cigarette smoke cause hyperplasia with pleomorphism of the newly formed cells in the bronchial epithelium of human foetal lung (Lasnitzki 1956, 1958) and benzopyrene, methycholanthrene and DMBA induce similar changes in rat tracheal epithelium (Crocker, Nielsen and Lasnitzki, 1965). Recently, it has become possible to concentrate the hydrocarbons in the cigarette smoke condensate (Whitehead, personal communication) and the action of this particular fraction has been examined in organ cultures of human foetal lung (Lasnitzki, 1968); it produces extensive basal cell hyperplasia with atypical cytological changes in all treated explants.

In experiments *in vivo* smoke or smoke condensates are being tested on rodents, mainly mice, and the results extrapolated to man. It seemed important to reverse the procedure and to compare the effects of the hydrocarbon enriched fraction already obtained on human lung *in vitro* with its action on the respiratory tract of the mouse grown under identical conditions. It was decided therefore to study the response of the mouse trachea to this fraction and results of the experiments are described in this paper.

The trachea was chosen for this investigation because it is known to react to carcinogenic hydrocarbons (Crocker *et al.*, 1965) and any results obtained on this material would provide a valuable comparison with the previous data on human explants.

### MATERIAL AND METHODS

The tracheas were derived from C3H mice either immediately before term or one day after birth. The organs were removed under aseptic conditions and divided into two parts, one to serve as control and the other as the experimental explant. The halves, each measuring approximately 3 mm. in length and 2 mm. in width were explanted as intact tubes and grown on strips of rayon acetate (Shaffer, 1954) by a modified Trowell technique (Trowell, 1959). Two of these strips, each carrying 3 explants were arranged on grids of stainless steel and placed in small culture chambers of borosilicate glass; the chambers were filled with medium, usually 2·5 ml., up to the level of the raft. Two chambers were enclosed

* Sir Halley Stewart Fellow.

in a Petri dish carpeted with 3 layers of damp filter paper to prevent evaporation. To reduce the risk of bacterial contamination the rayon strips carrying the explants were changed once a week to freshly sterilized grids, culture chambers and Petri dishes.

The Petri dishes were stacked in a Macintosh jar. After explantation and after each transfer the jar was perfused with a gas mixture of 95% oxygen and 5% $CO_2$ at a flow rate of 125 ml./minute for 30 minutes. This produced a concentration of 65% $\pm$ 2% oxygen inside the jar as measured by New's method (New, 1966).

The medium consisted of Parker's 199 with 20% heat inactivated horse serum and 2% chick embryo extract. Every 3–4 days it was sucked off with a pipette and replaced by fresh, medium leaving the explants undisturbed.

The hydrocarbon enriched fraction was provided by Dr. Day and Dr. Whitehead of the Tobacco Research Laboratories, Harrogate. The fraction which represents approximately 8% of the whole smoke condensate was dispersed under sterile conditions in calf serum with a high speed M.S.E. homogeniser. The smoke condensate was added to the medium in a concentration of 130 $\mu$g./ml. The explants were grown in this medium for 2 weeks, one group was then transferred to normal medium without the condensate for a further 3 days.

After 9, 14 and 17 days of cultivation, control and treated explants were fixed in 3% acetic Zenker's or in Bouin's solution, dehydrated, embedded in paraffin and sectioned at 6 $\mu$. The sections were stained with haematoxylin-eosin or by the periodic acid Schiff technique (PAS) after diastase digestion.

Mitotic and resting cells were counted in the tracheal epithelium before explantation and in control and treated explants after the various periods of cultivation. The mitotic index was expressed as a percentage of the total count, and, for each point on the mitotic curve, counts were made in at least 4 explants and in each of these 1000 cells were enumerated.

### RESULTS

### *Controls*

The trachea of mice shortly before or one day after birth is lined by one layer of cuboidal or columnar epithelium with small round basal nuclei (Fig. 1). The epithelium shows little or no secretory activity and cilia are absent and the mitotic index is 0·32% (Fig. 9). A layer of fibroblasts and fibres separates the epithelium from the surrounding cartilage; this is fully differentiated and consists of a matrix in which typical mature chondrocytes are embedded.

After 9 days *in vitro* the cartilage remains unchanged but the stroma between epithelium and cartilage is contracted. The epithelium has become slightly taller, has developed short cilia, and exudes PAS positive secretory matter into the lumen; the nuclei have moved towards the centre of the cells and the basal region is now frequently occupied by a layer of reserve cells (Fig. 2). The mitotic index of the epithelium ranges from 0·21% after 9 days' to 0·37% after 17 days' growth. Taking the standard deviations into account (Fig. 9) these values show that the mitotic activity of the tracheal epithelium is similar *in vivo* and remains constant during 17 days in culture.

The structure and architecture of the trachea observed after 9 days *in vitro* does not change materially during the rest of the culture period (Fig. 5).

*Effects of cigarette smoke condensate*

In tracheal explants exposed to the condensate for 9 days, the basal reserve cells of the epithelium have multiplied and cause the epithelium to become folded so that it often projects into the lumen. In most regions the superficial cells next to the lumen have enlarged, their cilia are better developed than in the control explants and their secretory activity has increased (Fig. 3). In places, however, the epithelium consists of several layers of irregular basal cells without superficial secretory elements; such areas are adjacent to actively secreting epithelium and tracheal glands (Fig. 4). With further exposure to the condensate, continued proliferation of the reserve cells causes a well marked epithelial hyperplasia. The basal cells enlarge irregularly and their nuclei often show crenated margins and prominent nucleoli (Fig. 7). After 2 weeks the metaplastic changes have involved the entire epithelium. Secretory activity has ceased and the superficial secretory cells have collapsed and been shed into the lumen; the presence of pycnotic nuclei in these dead cells indicate a rapid breakdown rather than a slow degeneration (Fig. 6). The secretory elements have been replaced by flat non-secretory cells with their long axes parallel to the lumen of the trachea.

The mitotic acitvity of the epithelium has risen to three times that of the controls after 9 days, and this increase is maintained at the same ratio throughout the whole of the 17-day culture period.

Although the condensate enhances epithelial proliferation it has a deleterious effect on the cartilage. After 2 weeks the chondrocytes have degenerated into pale ghosts in a matrix that has lost its staining power (Fig. 7). The necrotic cartilage becomes repopulated by immigrating fibroblasts from the connective tissue lying between the epithelium and the cartilage (Fig. 6).

The increased mitotic rate, hyperplasia and enlargement of the basal cells and absence of secretory cells in the explants treated with the condensate for 2 weeks, persist after the tissue has been transferred to control medium for a further 3 days. Invasion of the dead cartilage by fibroblasts is more active after transfer (Fig. 8).

### DISCUSSION

The response of the tracheal explants to the hydrocarbon enriched fraction shows two distinct phases. In the first, increased proliferation of reserve cells is combined with enlargement of the secretory cells and enhanced secretory activity; in the second the secretory cells are lost and not replaced, the hyperplasia has become more extensive and the newly formed cells have undergone metaplastic and anaplastic changes.

These effects are similar to those observed in organ cultures of human foetal lung exposed to the same hydrocarbon-enriched (Lasnitzki, 1968) except that here the secretory epithelium persists longer and is intact even after the hyperplastic cells have undergone squamous metaplasia.

A comparison of the present results with the effects seen in human foetal lung treated with benzopyrene and in rat tracheal epithelium exposed to individual carcinogenic hydrocarbons, shows that with these agents, the first phase, i.e. stimulation of secretory activity, is usually brief or absent and that the secretory epithelium breaks down at an early stage. This seems to be the only difference in effect between the hydrocarbons in smoke and the purified carcinogenic hydro-

FIG. 9. Showing increase in mitosis in tracheal epithelium treated with smoke condensate.

### EXPLANATION OF PLATES

FIG. 1.—Section through the trachea of a day-old mouse before explantation, showing columnar epithelium with basal nuclei and cellular connective tissue between epithelium and cartilage. H. and E. × 440.

FIG. 2.—Section through similar trachea grown for 9 days in control medium, showing columnar epithelium with cilia and a narrow zone of connective tissue between epithelium and cartilage. PAS+, × 440.

FIG. 3.—Section through another explant from the same trachea as in Fig. 2 treated for 9 days with the hydrocarbon enriched fraction. Note tall secretory cells, well developed cilia, secretory matter and multiplication of reserve cells. PAS+. × 440.

FIG. 4.—Tracheal epithelium in explant treated for 9 days with the hydrocarbon enriched fraction, showing metaplastic epithelium in close proximity to intact secretory epithelium and secreting tracheal gland. PAS+. ×440.

FIG. 5.—Tracheal epithelium in explant grown for 17 days in normal control medium. Note columnar epithelium with well developed cilia and some reserve cells near the basement membrane. PAS+. ×440.

FIG. 6.—Tracheal epithelium from explant grown for 2 weeks in presence of the hydrocarbon enriched fraction. Note basal cell hyperplasia, loss of secretory epithelium and fibroblasts in underlying cartilage. PAS+. ×440.

FIG. 7.—Tracheal epithelium from another explant grown for 2 weeks with the hydrocarbon enriched fraction, showing irregularly enlarged cells, pycnotic secretory cells and severely damaged cartilage (c). PAS+. ×440.

FIG. 8.—Tracheal epithelium from explant grown for 2 weeks with the hydrocarbon enriched fraction and for 3 days in normal control medium. Note basal cell hyperplasia, pycnotic secretory cells and cartilage filled with fibroblasts. PAS+. ×440.

Lasnitzki.

Lasnitzki.

carbons used on their own, as both induce basal cell hyperplasia associated with pleomorphism.

In contrast to its enhancement of epithelial growth, the hydrocarbon fraction has a deleterious effect on the cartilage. The chondrocytes are irreversibly damaged and the matrix becomes repopulated by fibroblasts. Similar changes have been observed in the bronchial cartilage of human foetal lung after exposure to benzopyrene and smoke condensates (Lasnitzki, 1956, 1958, 1968), and also in the connective tissue of mouse and rat prostates treated *in vitro* with methylcholanthrene (Lasnitzki, 1951, 1964).

The similarity in the response of the tracheal epithelium of rodents and of human foetal lung both to purified carcinogenic hydrocarbons and to the hydrocarbons in cigarette smoke suggests that, in view of the short supply of human foetal material for experimental purposes, the trachea of mice and rats is a valuable and suitable object in which to investigate the action of cigarette smoke on respiratory tissues.

### SUMMARY

The effects of a hydrocarbon-enriched fraction from cigarette smoke on mouse tracheas grown *in vitro* for 17 days were studied.

In control medium the tracheal epithelium consisted of one layer of ciliated columnar secretory epithelium and a few basal reserve cells.

The explants were grown for two weeks in the presence of the fraction and some were then transferred to control medium for a further 3 days.

After 9 days' treatment secretory activity and multiplication of the reserve cells were enhanced.

After 2 weeks' exposure the secretory cells were shed and the increased reserve cell multiplication had caused extensive basal cell hyperplasia with pleomorphism of the newly formed cells.

Epithelial mitosis in the treated explants rose to three times that of the control value.

The changes produced by the condensate persisted after transfer to normal medium for 3 days.

The tracheal cartilage was irreversibly damaged by the fraction.

I would like to thank Professor Honor B. Fell D.B.E., F.R.S., for advice and criticism in the preparation of this manuscript, Mr. George Lenney, A.I.S.T., for the micrographs and the British Empire Cancer Campaign for Research for some financial support of the work.

### REFERENCES

Cooper, R. L. and Lindsey, A. J.—(1955) *Br. J. Cancer*, **9**, 304.

Crocker, T. T., Nielsen, B. I. and Lasnitzki, I.—(1965) *Envir. Hlth*, **10**, 240.

Lasnitzki, I.—(1951) *Br. J. Cancer*, **5**, 345.—(1956) *Br. J. Cancer*, **10**, 547.—(1958) *Br. J. Cancer*, **12**, 510.—(1964) *Cancer Res.*, **24**, 973.—(1968) *Cancer Res.*, in press.

New, D. A. T.—(1966) 'The Culture of Vertebrate Embryos. London (Logos Press. Academic Press), p. 10.

Shaffer, B. M.—(1954) *Exp. Cell Res.*, **11**, 244.

Trowell, O. A.—(1959) *Exp. Cell Res.*, **16**, 118.